# Clinical Features

**Published:** 1936

**Authors:** C. Bruce Perry

**Affiliations:** Professor of Medicine, University of Bristol; Assistant Physician, Bristol General Hospital; Physician, Children's Hospital


### The Bristol Medico-Chirurgical Journal

*" Scire est nescire, nisi id me*
*Scire alius sciret."*

AUTUMN, 1936.

### CARCINOMA OF THE BRONCHUS.*

### Clinical Features.

By C. BRUCE PERRY, M.D., F.R.C.P.,

*Professor of Medicine, University of Bristol;*
*Assistant Physician, Bristol General Hospital;*
*Physician, Children's Hospital.*

Twenty years ago the diagnosis of cancer of the lung was rarely made, yet now we find carcinoma of the bronchus by no means a rarity. Whether this apparent increase in the incidence of the disease is an actual increase or not I do not propose to discuss. The purpose of this paper is to attempt to determine the incidence of the various clinical symptoms. In order to do this the cases admitted to the medical wards of the General Hospital during the last eight years have been analysed, and it is interesting to note that the forty cases thus considered show almost

* Papers read at a discussion by the Society on Wednesday, March 11th, 1936.

exactly the same symptomatology as other series of cases which have been published.

Before dealing with the signs and symptoms the age and sex incidence must be considered. All series of reported cases show that there is a very marked incidence of the disease in males as compared with females, and in the forty cases there were only six females. The age incidence in our series was highest between 40 and 70, and thirty-two of the cases fell into this age period. Cases do, however, occur at earlier ages; our youngest case was 23, and the oldest 76.

One of the first things that strikes one in a consideration of these cases is the duration of symptoms before the condition is recognized. It is hoped that by this discussion we may stimulate interest in the disease, and that this may lead to earlier recognition. As in any case of cancer, it is essential that early diagnosis should take place if treatment is to be of any avail. In our series the average duration of symptoms was just over 10½ months, but, and this is the most important point, an average of 6½ months elapsed between the onset of symptoms and the recognition of the condition. One patient lived at least four years after the onset of symptoms, and had been admitted to a sanatorium and discharged as non-tubercular eighteen months before I first saw him. He finally died about two years after we recognized the disease. A biopsy performed by Mr. Scarff through a bronchoscope showed this growth to be a squamous-celled carcinoma, which may account for the long course.

Turning to symptoms, the most important and consistent of these are dyspnœa, cough, and pain in the chest.

I have analysed the symptoms into first and second, as far as was possible, in an attempt to discover what symptoms should lead to an early diagnosis of the disease. In 39 cases cough was the first symptoms in 15, associated in 9 cases with blood-stained sputum. It appears to be rare for any gross hæmoptysis to occur in these cases, but a little streaking of the sputum with blood is not uncommon, and is very important, especially when it persists.

The classical red-currant-jelly sputum is rarely seen. Pain in the chest was the first complaint in 11 cases. This is variously described, but is definitely not a "pleural pain," being a constant pain unrelated, as a rule, to breathing or cough. Dyspnœa occurred as a first symptom in 10 cases. So that either cough, pain, or dyspnœa was the first complaint in 35 out of 39 cases (one case noted pain and dyspnœa simultaneously). Loss of weight we believe to be fairly common, though it was noted only rarely in our cases, I think because it was not specifically inquired for. Yet some cases undoubtedly suffer no gross loss of weight until comparatively late in the course of the disease. The other early symptoms are interesting, but of no serious value to diagnosis. Laryngeal palsy occurs from involvement of the recurrent laryngeal nerve by the tumour mass, or more probably by glands at the hilum, and usually means a fairly extensive growth. The frequency of cerebral metastases in this disease is well recognized, and one case was admitted to hospital as a hemiplegia, and it was some weeks before abnormal signs were detected in the chest. Such cases are not very uncommon. Harvey Cushing has removed secondaries from the brains of two cases who survived five and seven years respectively.[1] One patient was admitted with signs of cardiac failure, and post-mortem showed that the growth had invaded the pericardium with resultant cardiac embarrassment and failure. This case was regarded as rather a "freak." Yet in a recent paper from America 9 per cent. of the patients present themselves with this symptomatology.[2]

The second symptom noted by the patient is again usually either cough, pain, or dyspnœa, but in our series pain was by far the commonest second symptom noted. It occurred as a rule about two months after the onset of cough or dyspnœa.

SYMPTOMS IN THIRTY-NINE CASES.

| | First Symptom. | Second Symptom. (Usually about two months later than first.) | Total Symptoms. |
|---|---|---|---|
| Cough .. .. .. .. .. .. | 15 | 4 | 21 |
| Cough, with blood-stained sputum .. .. .. .. | (9) | (1) | (11) |
| Pain .. .. .. .. .. .. | 11 | 14 | 28 |
| Dyspnœa .. .. .. .. .. | 10 | 6 | 19 |
| Loss of weight .. .. .. | 3 | 3 | 8 |
| Laryngeal palsy .. .. .. | 2 | — | 2 |
| Cerebral symptoms .. .. | 1 | 1 | 3 |
| Cardiac failure .. .. .. | 1 | — | 1 |
| Dysphagia .. .. .. .. | — | — | 5 |

Taking the symptoms as a whole, 28 of the 39 cases complained of pain in the chest, 21 of cough, and 19 of dyspnœa. These, then, are the symptoms which must make us consider the possibility of carcinoma of the bronchus.

Turning to physical signs, these vary considerably according to whether there is any obstruction of the lumen of the bronchus by the growth, and whether this is partial or complete. In addition, pleurisy with effusion may develop, and, in fact, pleurisy apparently primary in any patient over 40 should immediately arouse a suspicion of carcinoma of the bronchus, but this as a rule is a late manifestation. A pleural effusion was present in 16 of our 40 cases when first examined, in 9 it was a clear effusion, in 6 hæmorrhagic, and in one purulent. Hæmorrhagic pleural effusions are, of course, very suggestive of malignant disease, but as you see the majority of malignant cases have a clear fluid. Complete obstruction of the lumen of the bronchus results in collapse of the lung beyond with flattening of the chest, diminished movement, impaired resonance, and diminished breath sounds. These signs were present in 10 cases when first seen. It is this diminished aeration and collapse which is responsible for the usual X-ray picture. Partial obstruction leads to the development of bronchiectasis in the lung peripheral to the growth, with clubbed fingers, foul sputum, and the physical signs of bronchial dilatation. 6 cases presented themselves with this picture. The tumour may present signs due to pressure on surrounding organs or as a result of its own bulk. Only 4 cases showed venous obstruction from pressure on veins—not nearly so frequently as in mediastinal tumours—three produced œsophageal obstruction, and one case produced physical signs of a solid tumour in the chest. Only 1 case showed no abnormal physical signs. But if radical surgical treatment is to have any real chance of success we must aim at diagnosing our cases at this stage. It must be admitted, however, that the early diagnosis of carcinoma of the bronchus is fraught with difficulty, especially as the very first symptom may be due to the development of metastases.

REFERENCES.

[1] Fried, B. M., *Medicine*, 1931, 10, 375.

[2] Arkin, A., and Wagner, D. H., *Journ. Amer. Med. Assoc.*, 1936, 106, 587.

